# A Lithium-ion Battery Using Partially Lithiated Graphite Anode and Amphi-redox LiMn_2_O_4_ Cathode

**DOI:** 10.1038/s41598-017-14741-x

**Published:** 2017-11-01

**Authors:** Yuju Jeon, Hyun Kuk Noh, Hyun-Kon Song

**Affiliations:** Department of Energy Engineering, School of Energy and Chemical Engineering, UNIST, Ulsan, 44919 Korea

## Abstract

Delithiation followed by lithiation of Li^+^-occupied (n-type) tetrahedral sites of cubic LiMn_2_O_4_ spinel (LMO) at ~4 ${{\bf{V}}}_{{{\bf{Li}}{\boldsymbol{/}}{\bf{Li}}}^{{\boldsymbol{+}}}}$ (delivering ~100 mAh g_LMO_^−1^) has been used for energy storage by lithium ion batteries (LIBs). In this work, we utilized unoccupied (p-type) octahedral sites of LMO available for lithiation at ~3 ${{\bf{V}}}_{{{\bf{Li}}{\boldsymbol{/}}{\bf{Li}}}^{{\boldsymbol{+}}}}$ (delivering additional ~100 mAh g_LMO_^−1^) that have never been used for LIBs in full-cell configuration. The whole capacity of *amphi*-redox LMO, including both oxidizable n-type and reducible p-type redox sites, at ~200 mAh g_LMO_^−1^ was realized by using the reactions both at 4 ${{\bf{V}}}_{{{\bf{Li}}{\boldsymbol{/}}{\bf{Li}}}^{{\boldsymbol{+}}}}$ and 3 ${{\bf{V}}}_{{{\bf{Li}}{\boldsymbol{/}}{\bf{Li}}}^{{\boldsymbol{+}}}}$. Durable reversibility of the 3 V reaction was achieved by graphene-wrapping LMO nanoparticles (LMO@Gn). Prelithiated graphite (Li_n_C_6_, n < 1) was used as anodes to lithiate the unoccupied octahedral sites of LMO for the 3 V reaction.

## Introduction

The global market size of lithium ion batteries (LIBs) is dramatically increasing as its application is expanding to electric vehicles and energy storage systems. Needs for high energy densities should be emphasized more than ever before in the large-scale systems. Energy densities of LIBs mostly depend on specific capacities of cathode active materials. Practical cathode materials of LIBs are mainly based on oxidation and reduction of transition metals (Co, Mn, Ni and Fe). Unfortunately, specific capacities of widely used transition-metal-based cathode materials are limited to ~100 mAh g^−1^ for LiMn_2_O_4_ in spinel (LMO), ~150 mAh g^−1^ for LiNi_x_Co_y_Mn_z_O_2_ in layered structure (x + y + z = 1; NMC) and < 170 mAh g^−1^ for LiFePO_4_ in olivine structure (LFP). Possibilities for reaching higher energy densities are supported by under-developing alternatives. Nickel-rich NMC (~180 mAh g^−1^ at x > 0.6 in LiNi_x_Co_y_Mn_z_O_2_)^1^ and overlithiated oxides (OLO; *x* Li_2_MnO_3_∙(1 − *x*) LiMO_2_ where M = Ni, Co and Mn; ~200 mAh g^−1^ at > 4.5 V)^2^ are the most popular candidates that battery makers are interested in. Recently, we reported that the electrochemistry of Mn^4+/3+^ of LMO around 3 V (Li_y_[Mn^3+^_y_Mn^4+^_2−y_]O_4_ at 1 < y < 2 in mixed cubic/tetragonal phase; equation 1 below) was possibly utilized in addition to the 4 V reaction of Mn^3+/4+^ used in LIBs (Li_y_[Mn^3+^_y_Mn^4+^_2−y_]O_4_ at 0 < y < 1 in cubic phase; equation 2 below) by nanosizing and wrapping LMO with a-few-layer graphene skin (LMO@Gn)^3^.

LiMn_2_O_4_ as a p-type (intrinsically oxidized) cathode around 3 V:

1 $${{\rm{LiMn}}}^{3+}{{\rm{Mn}}}^{4+}{{\rm{O}}}_{4}+x{{\rm{Li}}}^{+}+x{{\rm{e}}}^{-}={{\rm{Li}}}_{1+x}{{{\rm{Mn}}}^{3+}}_{1+x}{{{\rm{Mn}}}^{4+}}_{1-{\rm{x}}}{{\rm{O}}}_{4}\,\,$$

LiMn_2_O_4_ as an n-type (intrinsically reduced) cathode around 4 V:

2 $${{\rm{LiMn}}}^{3+}{{\rm{Mn}}}^{4+}{{\rm{O}}}_{4}={{\rm{Li}}}_{1-x}{{{\rm{Mn}}}^{3+}}_{1-x}{{{\rm{Mn}}}^{4+}}_{1+{\rm{x}}}{{\rm{O}}}_{4}+x{{\rm{Li}}}^{+}+x{{\rm{e}}}^{-}\,\,\,$$

Principally, more than 200 mAh g^−1^ can be extracted from LMO@Gn by using both electrochemical reactions.

In the practical configuration of LIB cells, lithium ions in n-type cathode materials are utilized for lithiating anode materials during charging: e.g., LiMn_2_O_4_ → Mn_2_O_4_ + Li^+^  + e^−^ in cathode and at the same time Li^+^  + e^−^ + C_6_ → LiC_6_ in graphite as an anode. In other words, reduced species, the reduction potential of which is relatively more positive to that of anode, are required for cathode materials. From a viewpoint of practical usages, however, the Mn^4+^ of LMO in the above-mentioned 3 V electrochemistry cannot be paired with p-type anodes such as bare graphite because LMO at 3 V is the p-type cathode that cannot be oxidized during the initial charging processes. To use the non-lithiated active materials as cathode materials, additional lithium should be provided to anode sides or lithium metal should be used as anodes. Lithium metal has been adopted as anodes for the oxygen and sulfur cathodes even if dendrite growth problems were not yet solved^4–6^. Moreover, LMO has the amphi-redox characteristics where both oxidation and reduction are possible (equations 1 and 2) due to its mixed oxidation number (Mn^4+^ and Mn^3+^) so that both lithium acceptor and donor are required in anodes.

If it were better not to use lithium metal as anode for the amphi-redox LMO, the use of prelithiated anode materials would be the answer. The partially or totally lithiated materials can be obtained by contacting host materials with lithium metal powder directly (or short-circuiting them)^7–11^. The very negative reduction potential of lithium metal drives lithium oxidation and host material reduction at the same time. The host materials are lithiated via intercalation or alloying reactions to keep charge neutrality. The prelithiation of anode is preferred because the cathode prelithiation might yield safety problems with excessive generation of heat of reaction. The prelithiation of graphite and silicon as anode materials has been reported. In addition to the lithiation^7,12–14^, the solid-electrolyte interphase (SEI) layer is pre-formed during the short-circuit prelithiation process so that a portion of electricity does not need to be consumed for the electrolyte decomposition during the first electrochemical charge^15–18^. Therefore, high initial coulombic efficiencies are guaranteed by the prelithiation.

In this work, we demonstrate the first case of the amphi-redox cathode material in full cell operation. The extra amount of lithium required for lithiating LiMn_2_O_4_ (amphi-redox species based on Mn^3+^ and Mn^4+^ half and half) to Li_2_Mn_2_O_4_ (fully reduced species based on only Mn^3+^) was supplied from pre-lithiated graphite (Li_*n*_C_6_ where *n* < 1). ~200 mAh g_LMO_^−1^ was reached by the use of amphi-redox LMO@Gn with the Li_*n*_C_6_.

## Results

The 4 V electrochemistry of LMO has been popularly used in commercial LIBs, its capacity (Q_4V_) reaching practically ~100 mAh g^−1^. Even if the theoretical value of the capacity for the 3 V reaction (Q_3V_) is equivalent to that of Q_4V_ at ~148 mAh g^−1^, it has been difficult to extract the 3 V capacity from practically used micrometer-size LMO particles: e.g., only 20 mAh g^−1^ was obtained for Q_3V_, which is much less than the theoretical value. In our previous work, we demonstrated that Q_4V_-level capacity (~100 mAh g^−1^) was also extracted from the 3 V reaction by nanosizing and wrapping LMO with a-few-layer graphene skin (LMO@Gn, Supplementary Fig. 1)^3^. ~200 mAh g^−1^ based on Q_4V_ plus Q_3V_ was realized in a *half* cell configuration of LMO@Gn||Li metal. In this case, the Li metal anode worked as an amphi-redox material.

**Figure 1:**
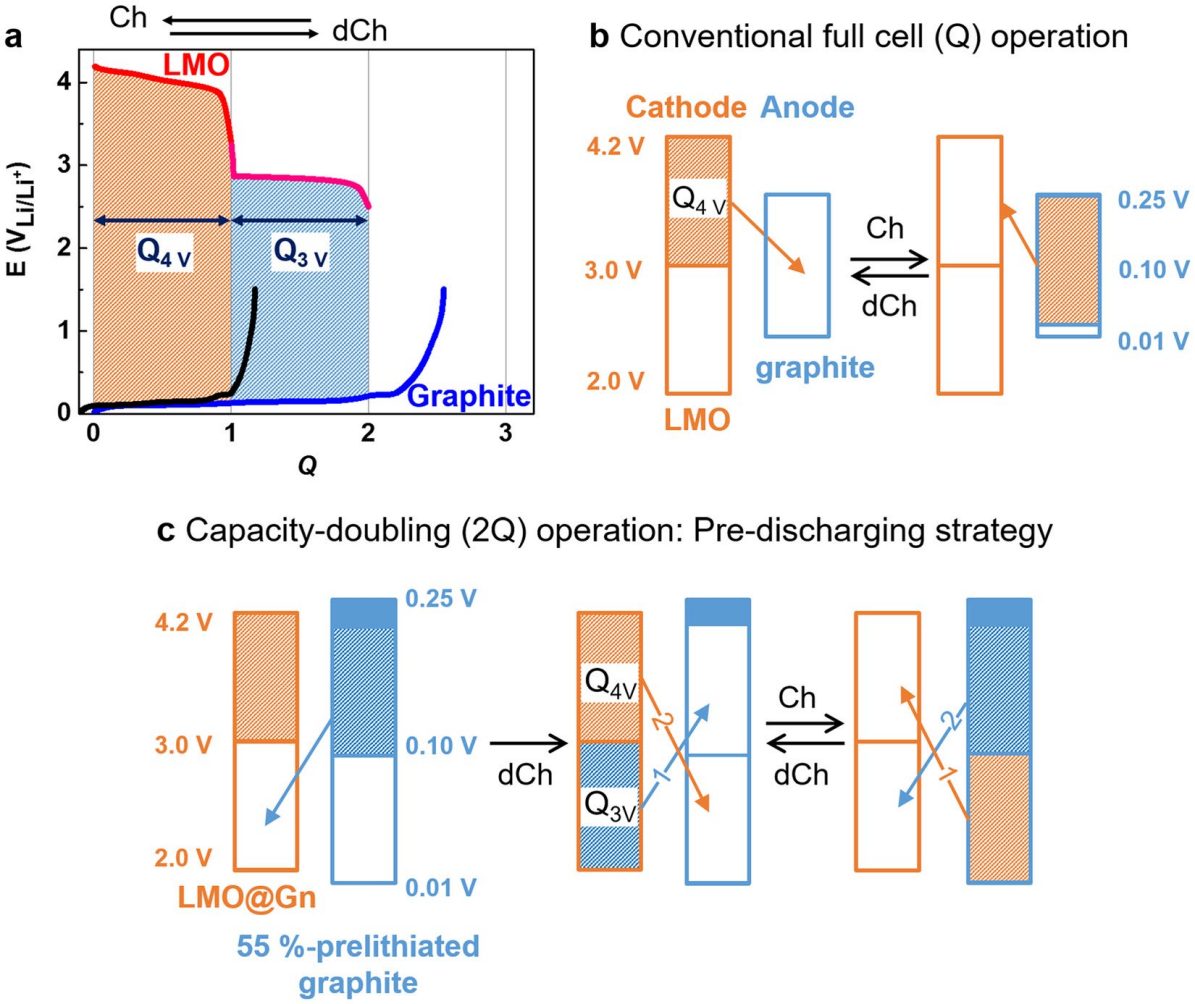
LMO@Gn||Li_*n*_C_6_ versus LMO||C_6_. (**a**) Potential (*E*) profiles of LMO (red + pink) and graphite (black and blue) along capacity (1Q = capacity of LMO at the practical 4 V reaction) for the conventional LMO||C_6_ (red versus black) and the LMO@Gn||Li_*n*_C_6_ (red + pink versus blue). (**b** and **c**) The operational schemes for the conventional LMO||C_6_ (**b**) and the LMO@Gn||Li_*n*_C_6_ (**c**). The capacity ratio of the anode to cathode (N/P ratio) = 1.10 for all cells. Ch = charge; dCh = discharge.

If the safety issues caused by the dendrite growth of Li^0^ on anodes were concerned, the amphi-redox LMO@Gn should be paired with an amphi-redox type anode such as partially lithiated graphite (Li_*n*_C_6_). The cell operation of LMO@Gn||Li_*n*_C_6_ is different from the conventional operation of LMO||C_6_ (Fig. 1). The lithium ions shuttle between the 4 V reaction region of LMO and the unlithiated empty region of graphite in LMO||C_6_ (Fig. 1a and b). The conventional operation of typical LMO was named Q operation here because the cell capacity is Q if Q_4V_ of LMO = Q. The conventional cells are initially in discharged states so that the cell operation is initiated by charge. On the other hand, the operation of the amphi-redox cells for doubling capacity (2Q operation) can be initiated by discharge as well as charge (Fig. 1c and Supplementary Fig. 2). In pre-discharging strategy of capacity-doubling operation (Fig. 1c), the empty 3 V reaction region of LMO@Gn is lithiated by lithium ions released from the prelithiated region of graphite to form Li_2_Mn_2_O_4_. The amount of lithium in the LMO@Gn becomes doubled so that its capacity is 2Q because Q_4V_ = Q_3V_. From the following charging and discharging processes, lithium ions move from fully lithiated LMO@Gn to fully unlithiated C_6_ during charging; and from fully lithiated C_6_ to fully unlithiated LMO@Gn during discharging (leaving 0.1Q in the upmost potential region lithiated at N/P = 1.1). The 4 V region of LMO@Gn is paired with the lower potential region of graphite (orange-colored) for lithium transfer while the 3 V region of LMO@Gn is paired with the higher potential region of graphite (blue-colored). In pre-charging operation (Supplementary Fig. 2), graphite is fully lithiated initially and then the following discharge/charge proceeds in the same way as the pre-discharging operation does.

**Figure 2:**
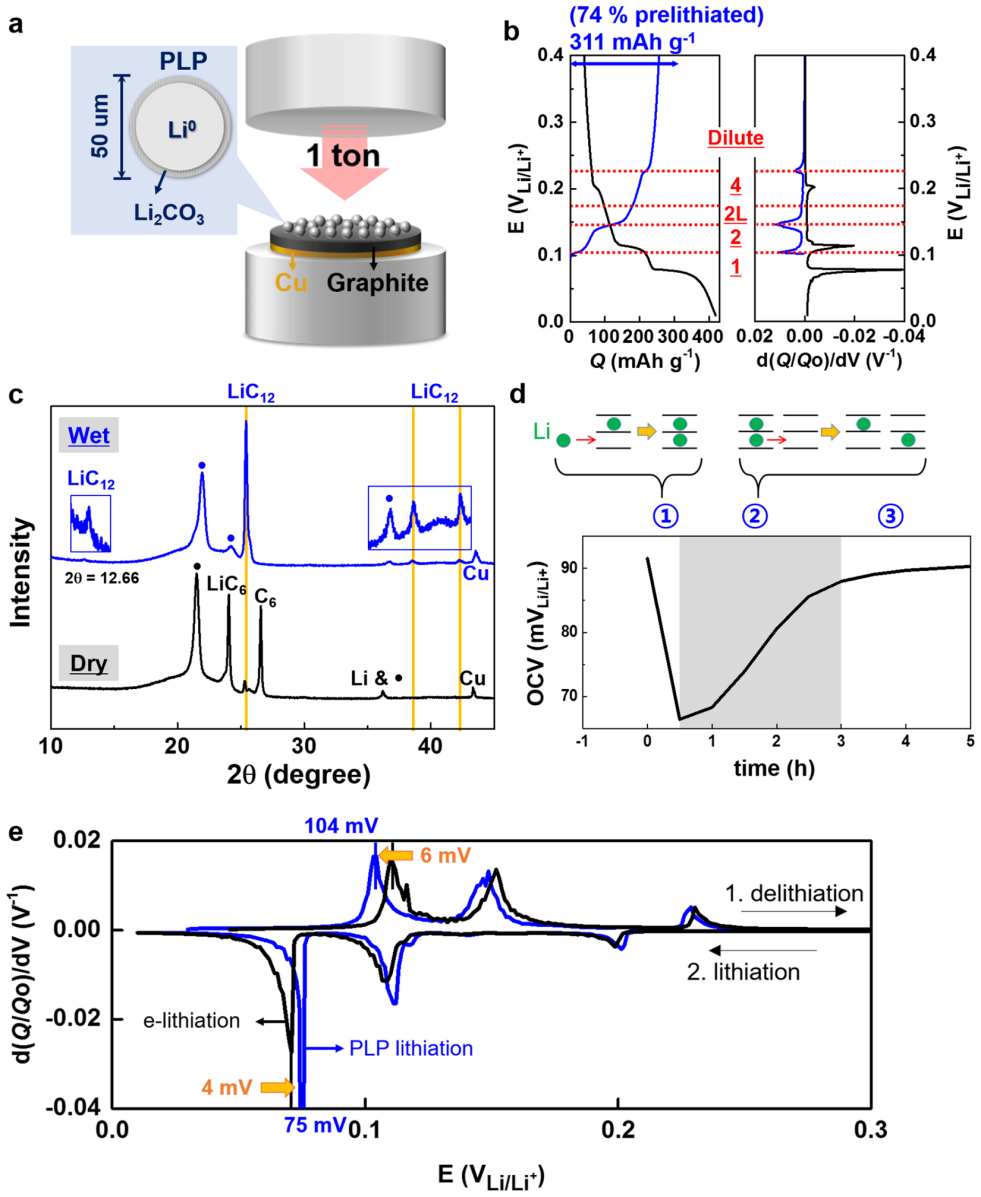
Prelithiated graphite (Li_*n*_C_6_, n < 1). (**a**) Press-activating PLP for prelithiating graphite anode. PLP = lithium metal powder surrounded by a passivated film of Li_2_CO_3_. The carbonate shell is broken by pressure at 1 ton so that a short circuit is developed between lithium metal and graphite. Electrons are transferred from lithium metal to graphite due to the reduction potential difference. At the same time, lithium ions generated as a result of the electron donation of Li^0^ are intercalated into the reduced graphite. (**b**) Potential profiles of 74 %-prelithiated graphite (*L*_PLP_ = 100 %) in Li_*n*_C_6_ (*n* = 0.74)||Li^0^ with respect to capacity (Q) or differential capacity (dQ/dV) along delithiation (blue) followed by lithiation (black). The stages of graphite intercalation compounds (GICs) were indicated by underlined red numbers or letters. (**c**) X-ray diffraction (XRD) patterns of lithiated graphite (Li_*n*_C_6_) before (black) and after (blue) being soaked in electrolyte. The peaks indicated by the black dots were assigned to a polymer film used for encapsulation to avoid the exposure to atmosphere. (**d**) Open circuit voltage (OCV) change of Li_*n*_C_6_||Li^0^. The numbers in circles indicate the three different OCV behaviors: drop-recovery-saturation. (**e**) dQ/dV curves of PLP-lithiated (blue) and electrochemically lithiated (black) graphites at the 20^th^ cycle at 0.2 C. The prelithiated graphites were delithiated and then lithiated in Li_*n*_C_6_||Li^0^.

Graphite anodes were prelithiated by pressing passivated lithium metal powder (PLP) loaded on graphite anodes by 1-ton hydraulic press in an Ar-filled glove box (Fig. 2a). Fractional conversion of loaded PLP to lithium available for delithiation (prelithiation yield, *Y*_Li_; refer to equation 3 in Methods for definition) was estimated at 65 to 75 % by the PLP sprinkle method. Lithiation of graphite by the press-activated PLP sprinkles was confirmed electrochemically and crystallographically. The potentials of the reaction plateaus and the corresponding dQ/dV peaks along deintercalation were consistent with the previously reported values^19–21^: LiC_6_ (stage 1) to LiC_12_ (stage 2) at 105 mV; LiC_12_ (stage 2) to LiC_18_ (stage 2L) at 141 mV; and LiC_36_ (stage 4) to LiC_72_ (dilute stage) at 223 mV (Fig. 2b and Supplementary Fig. 3 for 74 %-prelithiated graphite (Li_0.74_C_6_ = LiC_8_; *L*_PLP_ = 100 %); Supplementary Fig. 4a for over-prelithiated graphite obtained by loading excessive amount of PLP (*L*_PLP_ = ~700 %); Refer to equation 4 in Methods for the definition of *L*_PLP_). It indicates that lithium was intercalated to graphite framework by the PLP-prelithiation process. Also, the peaks of graphite intercalation compounds (GICs) at LiC_6_ and LiC_12_ were clearly found in the X-ray diffraction pattern of the same graphite electrode that is supposed to be lithiated by PLP^7,12,20,22^ (Fig. 2c; Supplementary Fig. 4b).

The distribution of lithium ions in graphite changed after the prelithiated graphite anodes were cured in electrolyte (Fig. 2c). Both LiC_6_ (major) and LiC_12_ (minor) were clearly found in the XRD pattern of the prelithiated graphite in the absence of electrolyte, indicating that lithium intercalation was localized. After curing in electrolyte for a day, on the contrary, the intensity of LiC_12_ peak dramatically increased and it was difficult to find LiC_6_ and C_6_ peaks. Therefore, we concluded that the intercalated lithium species were delocalized in the presence of electrolyte. The re-distribution of GICs caused by delocalization of lithium species in graphite was monitored by open circuit voltage (OCV) of half cells at Li_*n*_C_6_||Li^0^ (Fig. 2d). The OCV was dropped, recovered and then saturated. In a dry environment, only the grains or particles of graphite directly contacting lithium metal of PLP are lithiated to form LiC_6_ or LiC_12_ (only the two GICs were considered for simplicity). The rest of them are intact in unlithiated phase (C_6_). A portion of PLP is not used for lithiation, existing as a form of Li^0^. The potential difference is developed between the GICs and C_6_ and even between LiC_12_ and LiC_6_ throughout electrodes. As the electrodes consisting of C_6_, LiC_6_ and LiC_12_ are in contact with electrolyte, ionic pathways through electrolyte are developed between the lithiated and the unlithiated phases. The initial OCV drop indicates that C_6_ (unlithiated phase) and LiC_12_ (partially lithiated phase) are lithiated by lithium ions and electrons from Li^0^: Li^0^ + Li_x_C_y_ → Li_x+1_C_y_ (step 1 in Fig. 2d and Supplementary Fig. 5). The increase of lithiation amount results in the OCV drop. In the period of OCV increase following the drop, the lithiation delocalization is thought to proceed dominantly between GICs: e.g., 2LiC_6_ + 2C_6_ → LiC_12_ + LiC_12_ (step 2 in Fig. 2d and Supplementary Fig. 5). Less number of more intercalated GICs at more negative potentials are converted to more number of less intercalated GICs at more positive potential so that the OCV increases. After the lithiation delocalization is completed, the potential shows no more change (step 3 in Fig. 2d and Supplementary Fig. 5). This scenario is confirmed by two extreme cases (Supplementary Fig. 6). The initial OCV drop was not observed in a deficient PLP loading case where all Li^0^ is completely consumed for the lithiation. On the contrary, PLP overloading case showed only the OCV drop without the OCV recovery and saturation.

Interestingly, the prelithiated graphite anodes by PLP showed improved kinetics for electrochemical delithiation and the following electrochemical lithiation when compared with bare graphite. For strict comparison with the prelithiated graphite anode, the bare graphite electrode was pressed by the same pressure. The overpotentials of prelithiated graphite were lower than those of the bare graphite (Fig. 2e). For example, the first delithiation and the last lithiation peaks were observed at 104 mV and 75 mV in the PLP-prelithiated graphite, respectively (gap = 29 mV). The potential gap of the stage 1/stage 2 transition between delithiation and lithiation was 10 mV less than that of the bare graphite: peak-to-peak gap = 29 mV for PLP lithiation and 39 mV for electrochemical lithiation. Lithium metal conductor that was left after the PLP prelithiation would be partly responsible for the improved kinetics, the existence of which was supported by Li 1s X-ray photoelectron spectra (Supplementary Fig. 7). The reversibility of lithiation/delithiation of the prelithiated graphite was guaranteed (Supplementary Fig. 8).

Prelithiation starts from the highest potential at ~0.25 ${{\rm{V}}}_{{{\rm{Li}}/{\rm{Li}}}^{+}}$ and then the potential decreases along prelithiation. Therefore, the final potential of the GIC is determined by the extent of prelithiation of graphite (=*Y*_Li_ x *L*_PLP_ where *Y*_Li_ = ~70 %) depending on the loading amount of PLP. The capacity below the final GIC potential is used for accepting lithium coming from LMO at 4 ${{\rm{V}}}_{{{\rm{Li}}/{\rm{Li}}}^{+}}$. The minimum amount of prelithiation is 1Q used for lithiating LMO at 3 ${{\rm{V}}}_{{{\rm{Li}}/{\rm{Li}}}^{+}}$ during charging. Also, another 1Q should be guaranteed as the capacity of unlithiated phase of graphite ready for accepting lithium coming from LMO at 4 ${{\rm{V}}}_{{{\rm{Li}}/{\rm{Li}}}^{+}}$. By increasing the prelithiation amount above 1Q, extra lithium is intercalated into graphite. The lithium reservoir can compensate for the lithium deficiency caused by parasitic Li consumption reaction such as Mn deposition on anode (Mn^2+^ + 2LiC_6_ → Mn + 2Li^+^ + C_6_)^23–26^. However, the prelithiation amount should be limited not to drop the capacity of unlithiated graphite below 1Q. By increasing the amount of graphite at a fixed prelithiation amount, we can design the prelithiated graphite to have spare unlithiated capacity at lower potential as a safety margin for preventing lithium metal deposition. When the graphite amount increases from 2Q to 2.2Q at 1Q prelithiation, the capacity of unlithiated graphite increases from 1Q to 1.2Q (Supplementary Fig. 9a). Therefore, 0.2Q safety margin at lower potential is secured. In the same situation (graphite in 2.2Q) with higher prelithiation at 1.2Q (the prelithiation extent = 1.2Q / 2.2Q = 55 %), the potential during delithiation is kept at lower potential (potential gain) but the safety gain is lost (Supplementary Fig. 9b).

**Figure 3:**
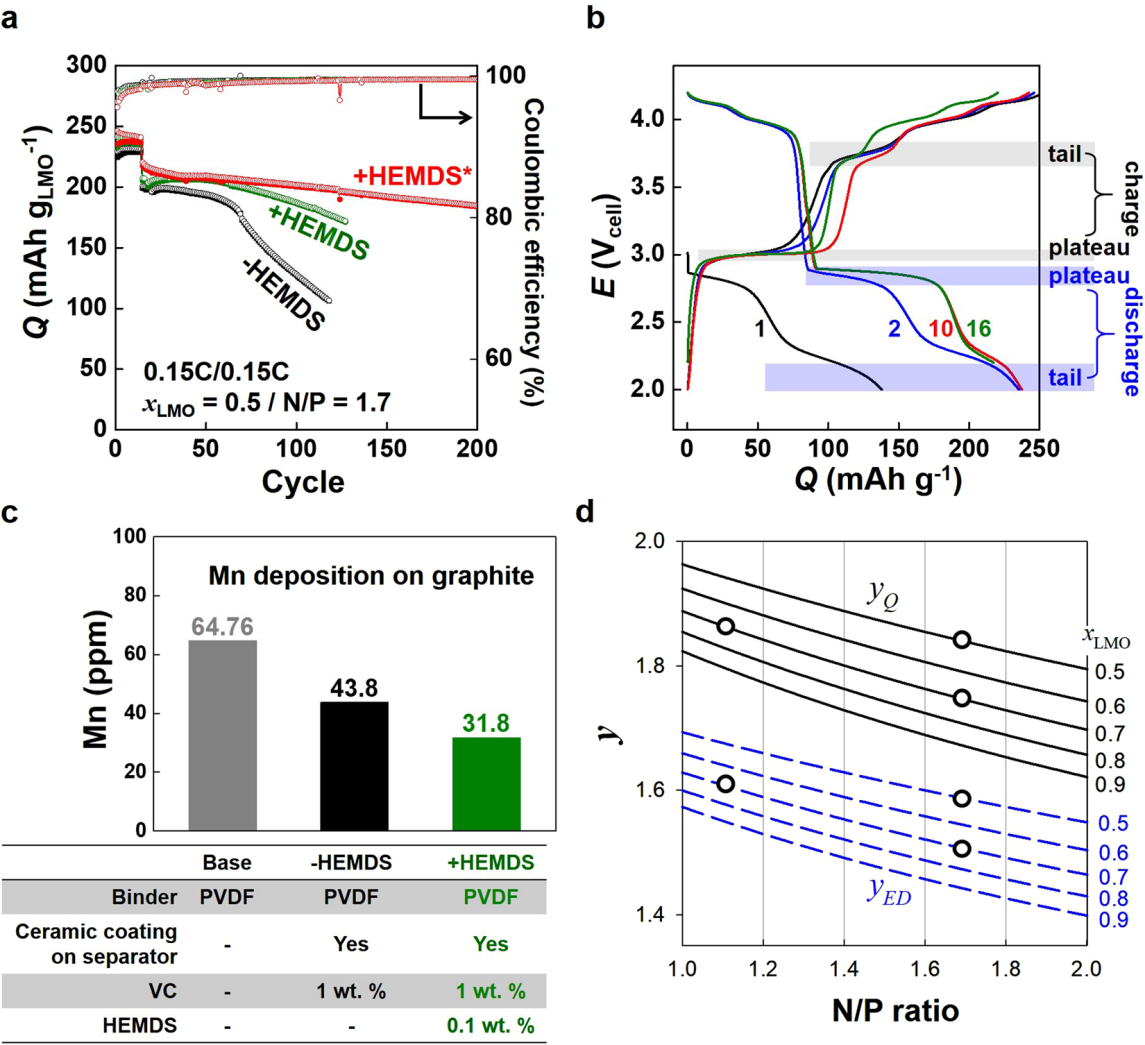
Amphi-LMO battery operation of LMO@Gn||Li_*n*_C_6_. +HEMDS (or +HEMDS*) and −HEMDS indicate cells containing HEMDS-present and HEMDS-absent electrolytes, respectively (HEMDS = heptamethyldisilzane). 0.1 wt. % HEMDS was used as a HF scavenger. Base electrolyte = 1 M LiPF_6_ in EC/EMC (1:2 v/v). 1 wt. % VC (vinylene carbonate) was used as an additive while ceramic-coated separator was used. PVDF was used as a binder for +HEMDS and −HEMDS while CMC was used for + HEMDS*. (**a**) Capacity retention and coulombic efficiency change along cycles at 0.1C for the initial 15 activation cycles in 2.0 V to 4.2 V followed by 0.15C in 2.2 V to 4.2 V. Open circle = Charge. Closed circle = Discharge. (**b**) Voltage profiles at selected cycles of +HEMDS* in (**a**). (**c**) Mn deposition on graphite anodes after repeated charge/discharge. Binder, separator and electrolyte additives used for battery cells were indicated. (**d**) Energy density improvement factor (*y*_ED_) and capacity improvement factor (*y*_Q_) as a function of the N/P ratio of LMO@Gn||Li_*n*_C_6_ (N/P) and the fractional content of LMO in cathodes (*x*_LMO_). LMO@Gn||Li_*n*_C_6_ and LMO||C_6_ were compared at the same condition of N/P and *x*_LMO_. The values of N/P and *x*_LMO_ used in this work were indicated by open circles.

The resultant prelithiated graphite anode realized the doubled capacity of LMO@Gn composed of Q_4V_ + Q_3V_ (+HEMDS* in Fig. 3a and Supplementary Fig. 10a for 50 wt. % and 70 wt. % LMO in cathodes, respectively). The capacity of the amphi-redox LMO batteries (LMO@Gn||Li_*n*_C_6_) was estimated at 200 mAh g_LMO_^−1^ based on LMO mass after the initial activation cycles. The capacity at the 100^th^ cycle was kept at > 90 % of the initial value after the 15 activation cycles with the capacity fade rate at ~0.2 mAh g^−1^ cycle^−1^ and the coulombic efficiency at ~100 %. The 70 wt. % LMO (equivalently, 76 wt. % LMO@Gn containing @Gn at 8.0 wt. % of LMO@Gn; Supplementary Fig. 10) in cathodes was considered as the Maginot line of the LMO amount. Only 4 wt. % margin was available for carbon black as conducting agent in the maximum LMO loading because 20 wt. % binder was required for the electrode integrity with LMO@Gn.

To secure the capacity of the 3 V reaction (Q_3V_) and its stability, interestingly, the activation process at low rate 0.1C during the initial 15 cycles was required. The lower cut-off cell voltage was lowered from 2.2 V_cell_ to 2.0 V_cell_ during the activation cycles. At the second cycle of the activation, Q_3V_ (excluding capacity based on the tail reaction nearby 2.0 V) was estimated only at ~50 mAh g_LMO_^−1^ that was ~50 % of the expected capacity. After the 10^th^ activation cycle, however, the 3 V plateau reaction (at ~2.85 V_cell_ for discharging and at ~3.0 V_cell_ for charging) increased to 100 mAh g_LMO_^−1^. At the same time, the tail reaction at 2.2 to 2.0 V_cell_ on discharge and at 3.65 to 3.8 V_cell_ on charge (Fig. 3b) decreased. The 3 V plateau reaction and the tail reaction were traded off while the sum of the two reactions was fixed at ~ 145 mAh g_LMO_^−1^ on discharge. After the activation cycle, the low cut-off voltage was set at 2.2 V_cell_ to secure the stability of the 3 V reaction.

It should be notified that the aforementioned cyclability of the amphi-redox cell of LMO@Gn||Li_*n*_C_6_ was guaranteed only after relieving problems of Mn dissolution of LMO@Gn (+HEMDS and +HEMDS* versus −HEMDS in Fig. 3a). The Mn dissolution problems of LMO have been well recognized, resulting in structural degradation of LMO and Mn deposition on anodes^23,24,27,28^. HF generated via the reaction between PF_6_^−^ and a trace amount of water in electrolyte drives the dissolution of Mn in a divalent cation form. The capacity decrease along cycle was much more serious in the full cell configuration of LMO@Gn||Li_*n*_C_6_ than that in the half cell configuration of LMO@Gn||Li^0^ (Supplementary Fig. 11). It is probably due to deposition of dissolved Mn^2+^ on anodes. Mn^2+^ is reduced to Mn^0^ while lithiated graphite is delithiated: 2LiC_6_ + Mn^2+^ → 2Li^+^ + 2C_6_ + Mn^0^. We implemented multiple strategies to suppress the Mn deposition. Alumina-coated separators were used to scavenge Mn^2+^ because the metal ions are adsorbed on alumina^29^. Na-carboxymethyl cellulose (CMC) was used as a binder for the same purpose since Mn ions are captured by ion exchange with Na ions^30^. 0.1 wt. % of heptamethyl disilazane (HEMDS) was introduced to electrolytes for scavenging HF which causes Mn dissolution^31,32^. Vinylene carbonate (VC) was used as an additive for making enhanced solid-electrolyte interphase (SEI) layer on anodes for preventing dissolved Mn^2+^ from penetrating through the SEI and then delithiating graphite^33^.

The amount of Mn metal detected on anodes after repeated charge/discharge cycles significantly decreased after adopting the former three strategies (Fig. 3c): from 65 ppm for the base electrolyte (without any additives); to 44 ppm after using the ceramic-coated separator and VC; moreover to 32 ppm when the HF scavenger was additionally adopted. CMC binder was reported to capture dissolved Mn^2+^ by exchanging Na^+^ with Mn^2+^ so that it possibly prevents capacity loss caused by Mn deposition^30^. As the results of these multiple Mn^2+^-blocking strategies, the capacity retention along cycle was successfully improved as shown in Fig. 3a from −HEMDS to + HEMDS to + HEMDS* (* = CMC). Capacity of LMO@Gn was completely recovered to 200 mA g_LMO_^−1^ at 0.15C even after rate capability tests at a series of C rate up to 5C (Supplementary Fig. 10c). Cathode morphologies did not change significantly along repeated cycles of charge and discharge (Supplementary Fig. 12).

## Discussion

The energy density (or capacity) gains obtained by using the double-capacity LMO in LMO@Gn||Li_*n*_C_6_ were determined by two factors: N/P and *x*_LMO_. N/P is the ratio of the capacity of anode to cathode in LMO@Gn||Li_*n*_C_6_. *x*_LMO_ is the LMO fraction in cathode. The energy density (or capacity) gains were quantified by the energy density (or capacity) improvement factor, *y*_ED_ (or *y*_Q_). The quantity was defined by the energy density (or capacity) ratio of LMO@Gn||Li_*n*_C_6_ to LMO||C_6_ at the same condition of N/P and *x*_LMO_ (Fig. 3d; equations 5 and 6 in Methods). Smaller N/P resulted in larger gains because saving extra graphite mass increased energy densities (or capacities) of the amphi-redox cells more than those of the practical cells based on only 4 V reaction. Smaller *x*_LMO_ (more amount of binders and conducting agents) decreased energy densities (or capacities) of both amphi-redox and practical 4 V cells but increased gains. The most important point is that the amphi-redox cells were superior to the practical cells over all N/P ratios in terms of energy density and capacity: *y*_ED_ = ~160 % (~60 % energy density gain) and *y*_Q_ = ~185 % (~85 % capacity gain) at N/P = 1.1. Also, the amphi-redox cells still showed higher energy densities and larger capacities in a cell level when compared with a practical 4 V cell containing 90 % LMO composition (*x*_LMO_ = 0.9, N/P = 1.1) (Supplementary Fig. 13). 25 % more energy density and 45 % larger capacity were achieved by the amphi-redox cells at the same N/P and 70 % LMO composition.

The amphi-redox cells were initially designed at N/P = 1.7 and *x*_LMO_ = 0.5 to maximize its capacity. Large amount of carbon black was used in cathode to compensate low conductivity of the tetragonal phase for the 3 V electrochemistry. However, the LMO content (*x*_LMO_) increase to *x*_LMO_ = 0.7 showed negligible change in cyclability (Supplementary Fig. 10a). Also, the high N/P secures a potential margin of lithium storage. In a typical full cell design for n-type cathode||p-type anode, on the other hand, the N/P is practically set at < 1.1. One of the limitations of the amphi-redox cells of LMO@Gn||Li_*n*_C_6_ is its low operational voltage. To use the 3 ${{\rm{V}}}_{{{\rm{Li}}/{\rm{Li}}}^{+}}$ reaction as well as the 4 ${{\rm{V}}}_{{{\rm{Li}}/{\rm{Li}}}^{+}}$ reaction, the cut-off potential of LMO@Gn is set at < 2.5 ${{\rm{V}}}_{{{\rm{Li}}/{\rm{Li}}}^{+}}$, which is lower than the cut-off potentials of practical LMO cells. Therefore, lower potential region of graphite at < 0.25 ${{\rm{V}}}_{{{\rm{Li}}/{\rm{Li}}}^{+}}$ should be utilized with 0.05 V margin to secure the cell voltage above 2.2 V_cell_. As the overpotential on LMO is developed along repeated charge/discharge cycles, the lower-potential region of graphite should be used. By increasing the N/P ratio to 1.7, 1.4Q below 0.25 ${{\rm{V}}}_{{{\rm{Li}}/{\rm{Li}}}^{+}}$ is reserved for lithium consumption caused by Mn deposition (1.4Q in Supplementary Fig. 14). When the N/P ratio decreased to the practical value 1.1, capacity retention of the cell at N/P = 1.1 became inferior to that of high N/P cells (N/P = 1.7) after the 50^th^ cycle (Supplementary Fig. 10b). The main reason for the deterioration is the loss of potential margin.

In conclusion, LIB cells based on amphi-redox active materials were presented, where the capacity of LiMn_2_O_4_ spinel (LMO) as a cathode material was doubled to be 200 mAh g^−1^ in full cell configuration. Conventionally, fully lithiated n-type and fully unlithiated p-type electroactive species have been used as cathode and anode materials of LIBs, respectively. LMO is a popular cathode material based on Li^+^-preoccupied (n-type) tetrahedral sites of cubic phase that is oxidized at ~4 ${{\rm{V}}}_{{{\rm{Li}}/{\rm{Li}}}^{+}}$ to release lithium ions in ~100 mAh g_LMO_^−1^ during charging. Even if LMO has additional 100 mAh g_LMO_^−1^ capacity based on unoccupied (p-type) octahedral sites of tetragonal phase at ~3 ${{\rm{V}}}_{{{\rm{Li}}/{\rm{Li}}}^{+}}$, it has been impossible to utilize the whole capacity at ~200 mAh g^−1^ in conventional cell and material designs due to instability of the 3 V reaction and the p-type nature of the 3 V active sites. We realized the whole capacity of the amphi-redox LiMn_2_O_4_ spinel in a mixed nature of n-type (4 V) and p-type (3 V). Reversible utilization of the 3 V reaction was made possible (1) by graphene-wrapped LMO nanoparticles (LMO@Gn) guaranteeing structural stability and electrical conduction and (2) by additional lithium source in prelithiated graphite (Li_n_C_6_, n < 1) to lithiate the unoccupied octahedral sites of tetragonal LMO.

## Methods

### LMO@Gn cathodes

A mixture of LMO (Aldrich, undoped) and graphite (Timcal KS6) at 80:7 w/w was ball-milled in a stainless vial by using a high-energy vibratory ball miller (SPEX 8000D) to yield LMO@Gn. A mixed slurry of LMO@Gn, carbon black (Timcal Super P) and polyvinylidene fluoride (PVDF; Solef 5130) in N-methyl-2-pyrrolidone was casted on the aluminum foil and dried at 120 °C for 1 h. The resultant mass composition of the cathode was fixed at 70 : 6.125 : 3.875 : 20 or 50 : 4.4 : 25.6 : 20 for LMO/Gn/carbon black/PVDF.

### Li_*n*_C_6_ anodes

A mixed slurry of natural graphite (Mitsubishi Chemical), carbon black (Timcal Super P) and a binder in N-methyl-2-pyrrolidone was casted on the copper foil and dried at 120 °C for 2 h. Na-carboxymethyl cellulose (CMC; Sigma-Aldrich) or polyvinylidene fluoride (PVDF; Solef 5130) was used as the binder. The resultant mass composition of the anode was fixed at 7 : 2 : 1 for graphite/carbon black/PVDF. The graphite anodes were lithiated by activating passivated lithium powder (PLP, China Energy Lithium Co., Ltd) sprinkles on active layers of anodes. The anodes, on which a proper amount of PLP was loaded, was pressed at 1 ton for 30 s by using a hydraulic press (Specac, AtlasTM manual 15-ton hydraulic press). Two parameters of prelithiation were defined as follows:

3 $${\rm{Prelithiation}}\,{\rm{yield}}={Y}_{{\rm{Li}}}( \% )=m/{{m}_{o}}^{* }100$$

where *m* = the amount of lithium intercalated to graphite during prelithiation process (g) = *Q* × (3.6 C / 1 mAh) / *n* F × MW_Li_; *m*_o_ = the amount of PLP loaded (g) with *Q* = delithiation capacity of graphite prelithiated by PLP (mAh); *n* = 1 mol e (mol Li)^−1^ for Li^+^  + e^−^ → Li^0^; F = Faraday constant = 96500 C (mol e)^−1^; MW_Li_ = 6.94 g (mol Li)^−1^ and

4 $$ \% {\rm{PLP}}\,{\rm{Loading}}={L}_{{\rm{PLP}}}={m}_{0}/{m}_{100}\times 100$$

where *m*_100_ = the amount of lithium required for fully lithiating graphite (g).

### Amphi-redox battery

LMO@Gn||Li_*n*_C_6_, LMO@Gn||Li^0^ and Li_*n*_C_6_||Li^0^ were realized in the 2032 coin cell configuration. Cells were assembled in a glove box of argon atmosphere (H_2_O < 0.1 ppm and O_2_ < 0.1 ppm). 1 M LiPF_6_ in EC/EMC (1:2 v/v; EC = ethylene carbonate, EMC = ethyl methyl carbonate) was used as a base electrolyte. 1 wt. % VC was used as an additive for electrolytes to form the solid-electrolyte-interphase (SEI) layer. 0.1 wt. % heptamethyl disilazane (HEMDS) was used as a HF scavenger. Used as a separator was porous polyethylene separator (Asahi) or two-sided-alumina-coated polyethylene separator (2 μm Al_2_O_3_/16 μm PE/2 μm Al_2_O_3_; W-Scope).

**Improvement factors (y**_**ED**_ **and y**_**Q**_ **)**.

5 $${y}_{{\rm{ED}}}=\frac{\,\frac{{Q}_{4}{V}_{4}+{Q}_{3}{V}_{3}}{\frac{1}{{x}_{LMO}}+\frac{{Q}_{4}+{Q}_{3}}{{Q}_{Gr}}NP}\,}{\frac{{Q}_{4}{V}_{4}}{\frac{1}{{x}_{LMO}}+\frac{{Q}_{4}}{{Q}_{Gr}}NP}}$$

6 $${y}_{Q}=\frac{\frac{{Q}_{4}+{Q}_{3}}{\frac{1}{{x}_{LMO}}+\frac{{Q}_{4}+{Q}_{3}}{{Q}_{Gr}}NP}}{\frac{{Q}_{4}}{\frac{1}{{x}_{LMO}}+\frac{{Q}_{4}}{{Q}_{Gr}}NP}}$$

where *y*_ED_ (*y*_Q_) = energy density (capacity) improvement factor = the ratio of energy density (capacity) of LMO@Gn||Li_n_C_6_ to LMO||C_6_; subscript 4 and 3 = 4 V and 3 V, respectively; Q = specific capacity; Q_4_ and Q_3_ = specific capacity of LMO at 4 V and 3 V, respectively; Q_Gr._ = theoretical capacity of graphite; V = voltage; NP = capacity ratio of anode to cathode in LMO@Gn||Li_*n*_C_6_; *x* = *x*_*LMO*_ = LMO fraction in cathode.

### Physicochemical characterization

Graphite intercalation compounds (GICs) in lithiated anodes were identified by X-ray diffraction (XRD; Bruker AXS, D8 Advance with Cu–Kα radiation). Lithiated graphite anodes in a circle shape were hermetically sealed by transparent polymer film used for vacuum packaging (Food Keeper, VP-9900) in a glove box to prevent oxidation. The hermetically sealed anode samples were placed on XRD sample holders (not inside the well for powder samples) and fixed on the rims of the holders by tape. The front positions of samples on which X-ray was incident were not perfectly identical over every run so that slight peak position shifts were observed (Supplementary Fig. 15). Lithium metal on lithiated graphite was identified by X-ray photoelectron spectroscopy (XPS) (ThermoFisher K-alpha). Deposited Mn on graphite anode was quantified by using optical emission spectroscopy where samples were atomized by inductively coupled plasma (ICP-OES; Varian, 700-ES). Graphite anode samples were obtained from cells experiencing electrochemical cycles, followed by washing by dimethyl carbonate (DMC) and then drying. Whole graphite anodes were used for quantifying Mn deposits in a cell.

## Electronic supplementary material

Supplementary information
