# Supplementary material for: A Lithium-ion Battery Using Partially Lithiated Graphite Anode and Amphi-redox LiMn2O4 Cathode
Source: Sci Rep. 2017 Nov 1;7:14879. doi: 10.1038/s41598-017-14741-x (PMC5666002; doi:10.1038/s41598-017-14741-x)
Supplement: Supplementary file 1 — Supplementary information [file 41598_2017_14741_MOESM1_ESM.doc]

Supplementary materials

**A Lithium-ion Battery Using Partially Lithiated Graphite Anode and Amphi-redox LiMn_2_O_4_ Cathode**

Yuju Jeon,^†^ Hyun Kuk Noh^†^,* and Hyun-Kon Song*

Department of Energy Engineering, School of Energy and Chemical Engineering, UNIST, Ulsan 44919, Korea

^†^ These authors contributed equally to this work.

^*^ Correspondence should be addressed to H.K.N. (email: hknoh21@gmail.com) or H.-K.S. (email: philiphobi@hotmail.com).


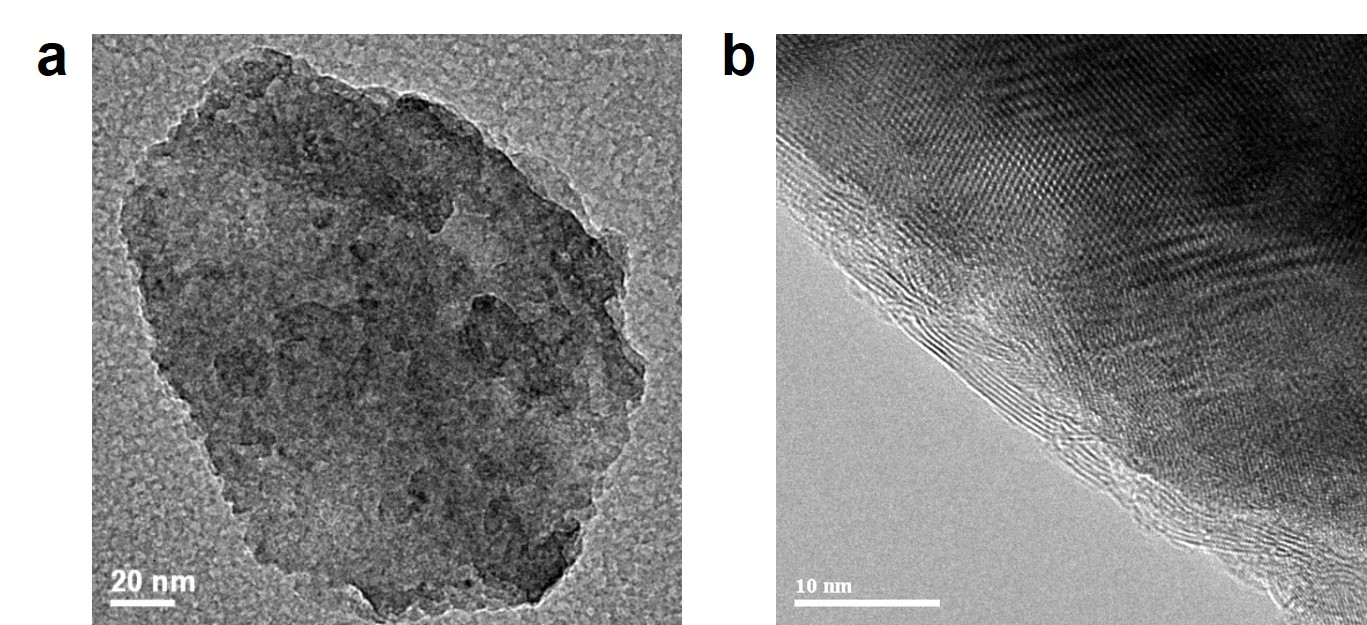


**Supplementary Figure 1.** TEM images of LMO@Gn. The a-few-layer graphene skin (@Gn) of LMO@Gn was observed in (**b**) that is magnified from (**a**).


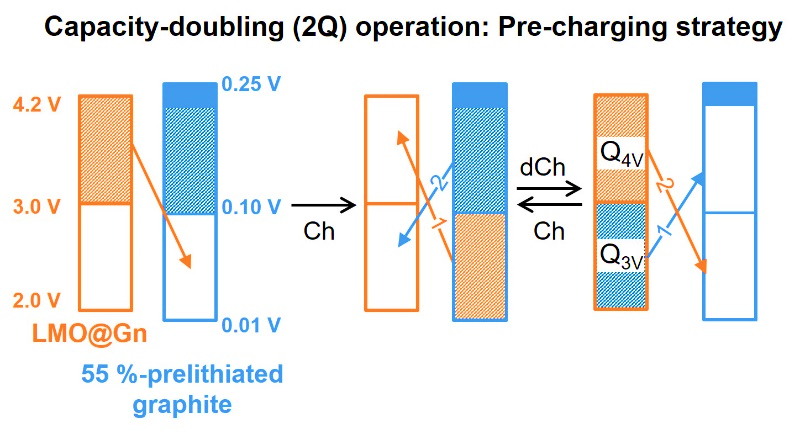


**Supplementary Figure 2.** The pre-charging strategy of the capacity-doubling operation for the LMO@Gn||Li*_n_*C_6_. The pre-discharging strategy was shown in Figure 1c.


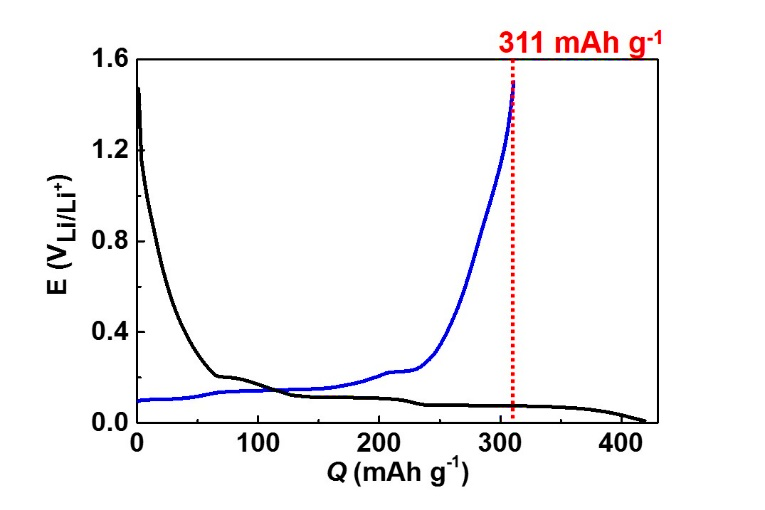


**Supplementary Figure 3.** Potential profiles of 74 %-prelithiated graphite (*L*_PLP_ = 100 %) in Li*_n_*C_6_ (*n* = 0.74) || Li^0^ with respect to capacity (Q) along delithiation (blue) followed by lithiation (black). Gravimetric capacity was calculated by considering only the mass of graphite. The capacity larger than the theoretical value of graphite (372 mAh g^-1^) was obtained during lithiation following delithiation because 20 % carbon black provided additional capacity. 74 % prelithiation was calculated by dividing the delithiation capacity (311 mAh g^-1^) by the lithiation capacity (420 mAh g^-1^).


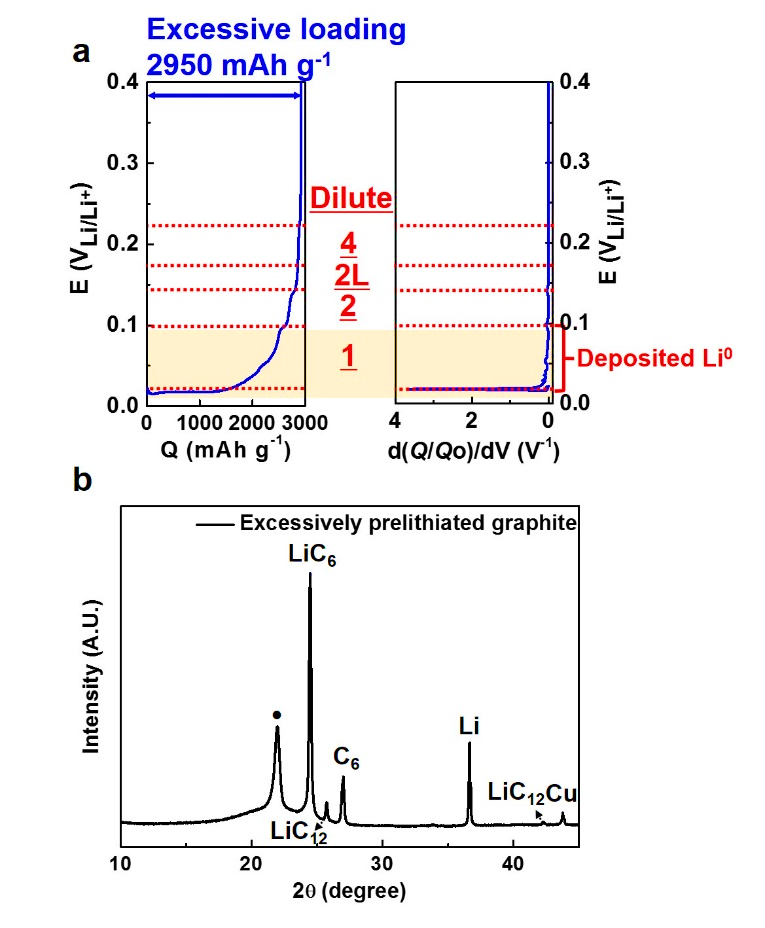


**Supplementary Figure 4. Over-prelithiated graphite anode obtained by loading excessive amount of PLP (*L*_PLP_ = ~700 %; Li*_n_*C_6_ (*n* > 1) or fully lithiated graphite (LiC_6_) + lithium metal (Li^0^)).** (**a**) Potential profile in the cell of Li*_n_*C_6_||Li^0^ (*n* > 1). (**b**) XRD pattern. The sample for XRD was not aged in electrolyte so that graphite intercalation compounds (GICs) was not re-distributed. The black dot indicates the polymer film used for encapsulation.


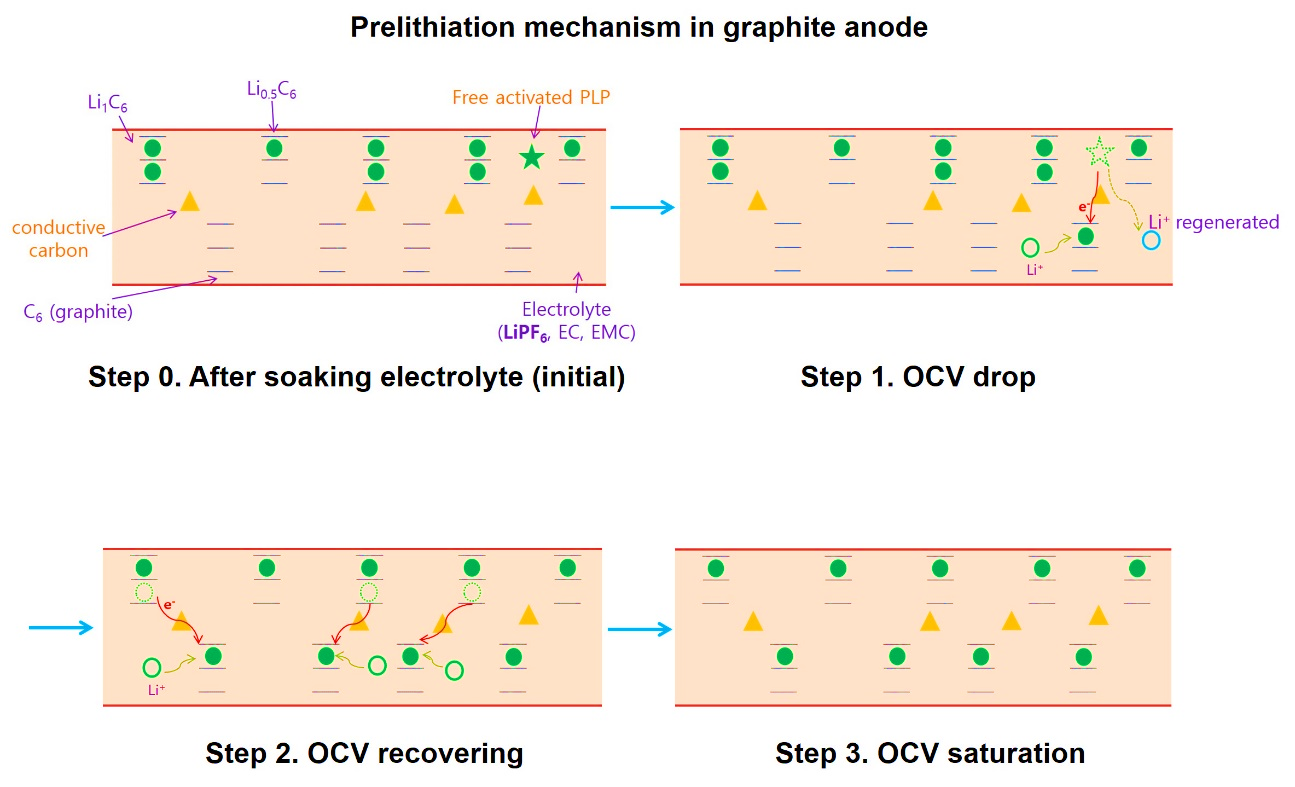


**Supplementary Figure 5.** Lithium re-distribution in prelithiated graphite in the presence of electrolyte.


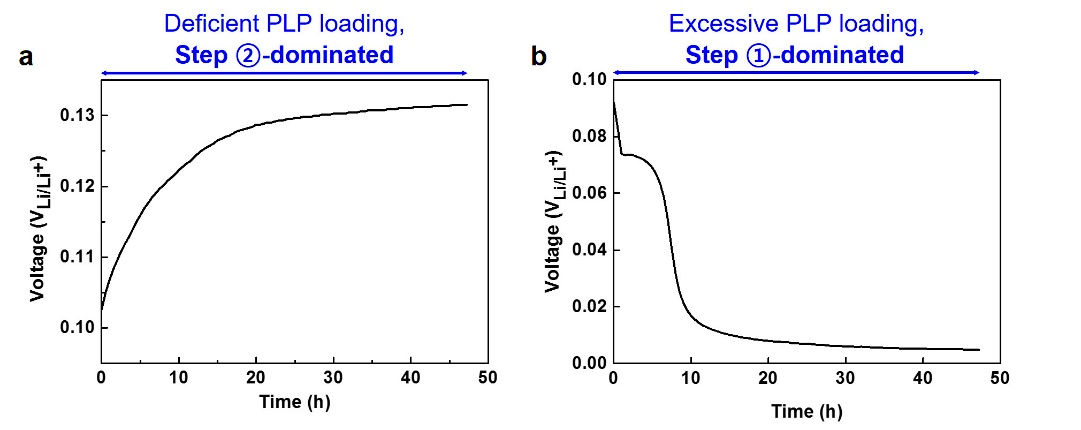


**Supplementary Figure 6.** OCV change of Li*_n_*C_6_||Li^0^ where n is (**a**) << 1 and (**b**) > 1.


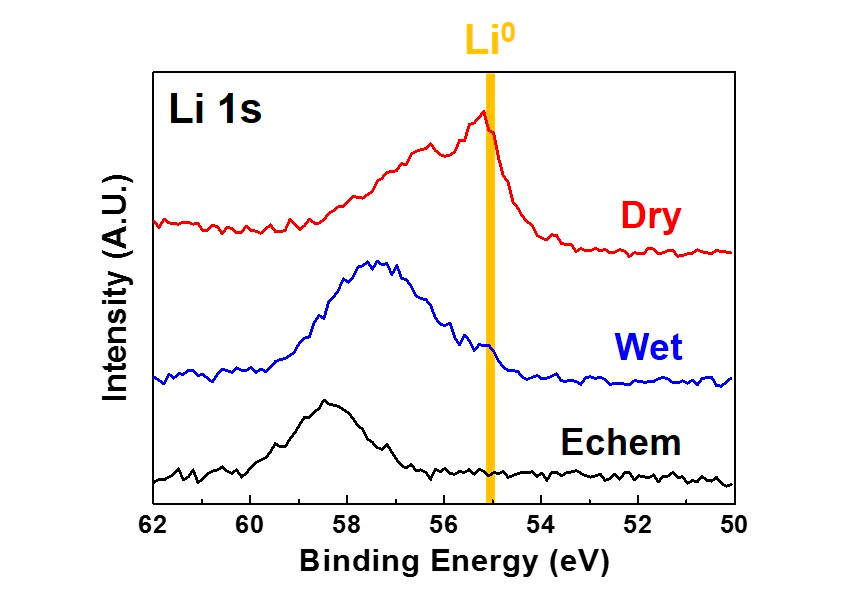


**Supplementary Figure 7.** Li 1s X-ray photoelectron spectra (XPS spectra) of lithiated graphites prepared by three different methods: **Echem** = electrochemically lithiated graphite; **Dry** = PLP-lithiated graphite before electrolyte aging; **Wet** = PLP-lithiated graphite after electrolyte aging. The peaks at 58.5 eV and 55.0 eV correspond to completely oxidized and metallic lithium, respectively. Samples were exposed to air for ~20 s before the XPS measurement. The exposure to air caused oxidation of metallic lithium. Therefore, lithium in the electrochemically lithiated graphite was found at 58.5 eV that is assigned to the fully oxidized state (Echem). On the contrary, a dominant population of lithium was less oxidized or closer to lithium metal (55 eV) in the PLP-lithiated graphite before aging in electrolyte (Dry). After electrolyte aging, a portion of lithium metal is thought to be oxidized, which is indicated by the peak shift from 55 eV to 57.3 eV (Wet). However, the peak of the PLP-lithiated graphite after electrolyte aging (Wet) was observed at the binding energy still less than that of the electrochemically lithiated graphite (EChem). Less oxidized states of lithium containing metallic lithium are expected in the PLP-lithiated graphite aged in electrolyte.


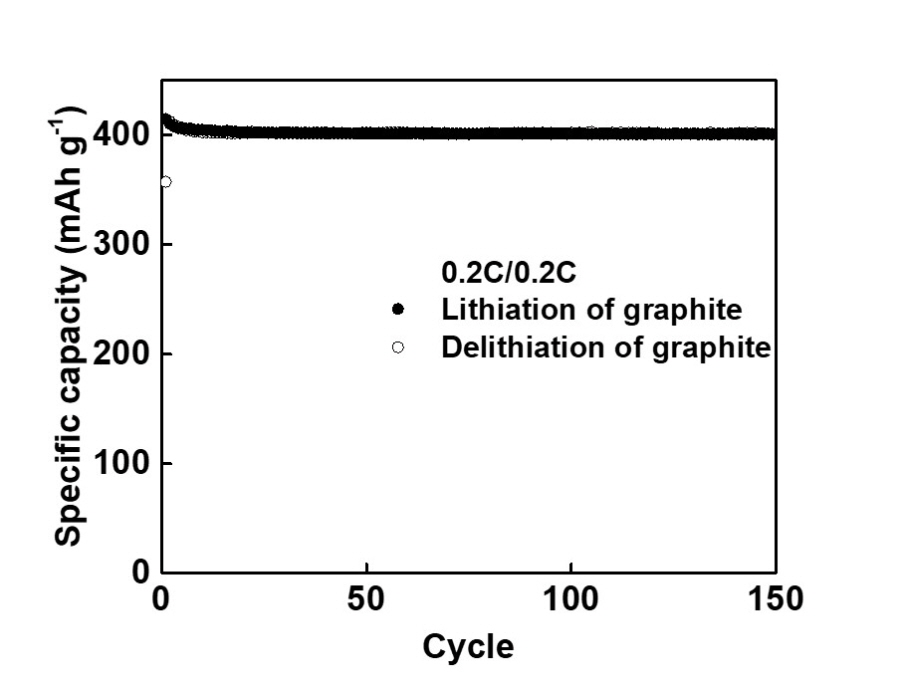


**Supplementary Figure 8.** Electrochemical stability of prelithiated graphite in a half cell. Capacities were calculated based on the mass of graphite. The capacity exceeded the theoretical capacity of graphite due to the reversible capacities of carbon black at ~200 mAh g^-1^.


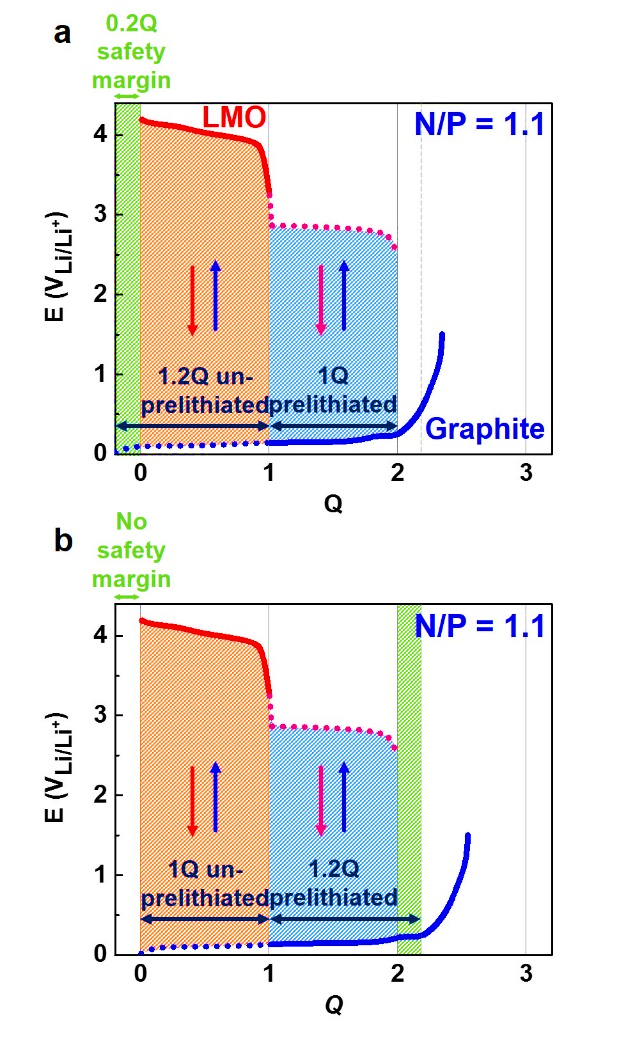


**Supplementary Figure 9.** **Operation design of** **LMO@Gn||Li_n_C_6_.** Potential (E) profile in the cases where (**a**) 1Q of lithium is prelithiated for remaining 0.2Q of safety margin (safety gain) and (**b**) 0.2Q is additionally prelithiated for securing cell potential (potential gain).


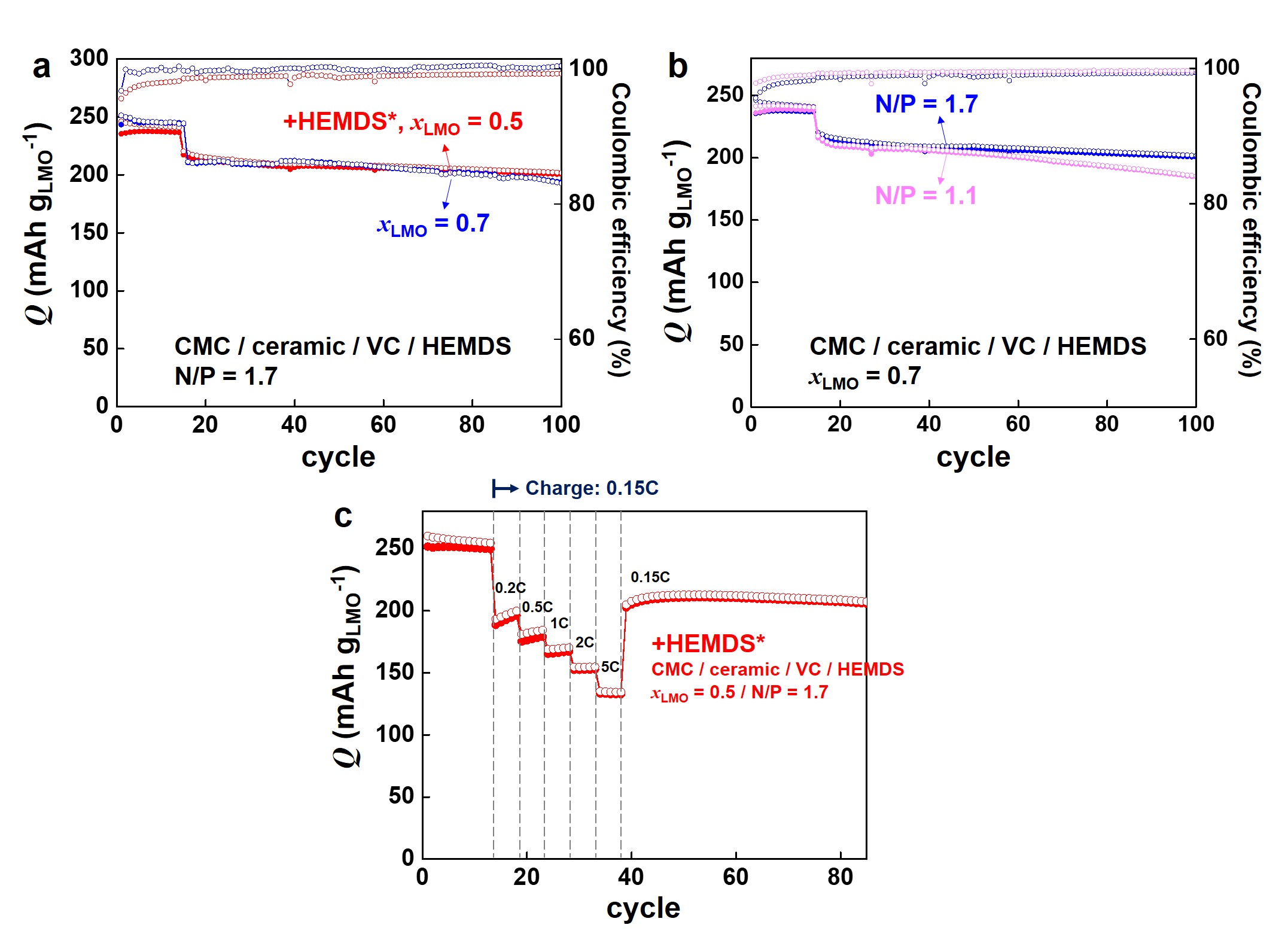


**Supplementary Figure 10.** **Cyclability and rate capability of LMO@Gn||Li_n_C_6_ at different conditions.** Detailed conditions of electrolyte, electrodes and separator were indicated. The LIB cells were charged and discharged at 0.15C for cyclability tests while they were charged at 0.15C and discharged at various C rates for rate capability tests. (**a**) Different compositions of LMO in electrodes (*x*_LMO_) at 0.7 versus 0.5 with N/P = 1.7 in +HEMDS*. (**b**) Different N/P ratios at 1.7 versus 1.1 with *x*_LMO_ = 0.7 in +HEMDS*. (**c**) Discharge capacities at various C-rates. The cell was charged at 0.15C after 15 cycles. The initial 15 cycles are for the activation process at 0.1C charge and discharge with the low cut-off at 2.0 V.


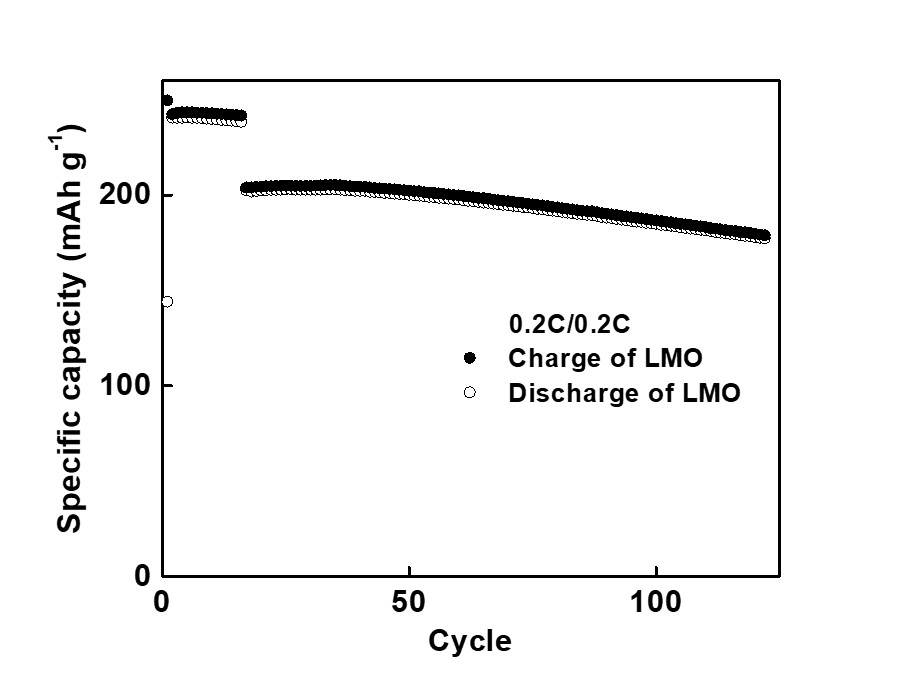


**Supplementary Figure 11.** Capacity retention of LMO@Gn in half cell along repeated cycles of charge/discharge.


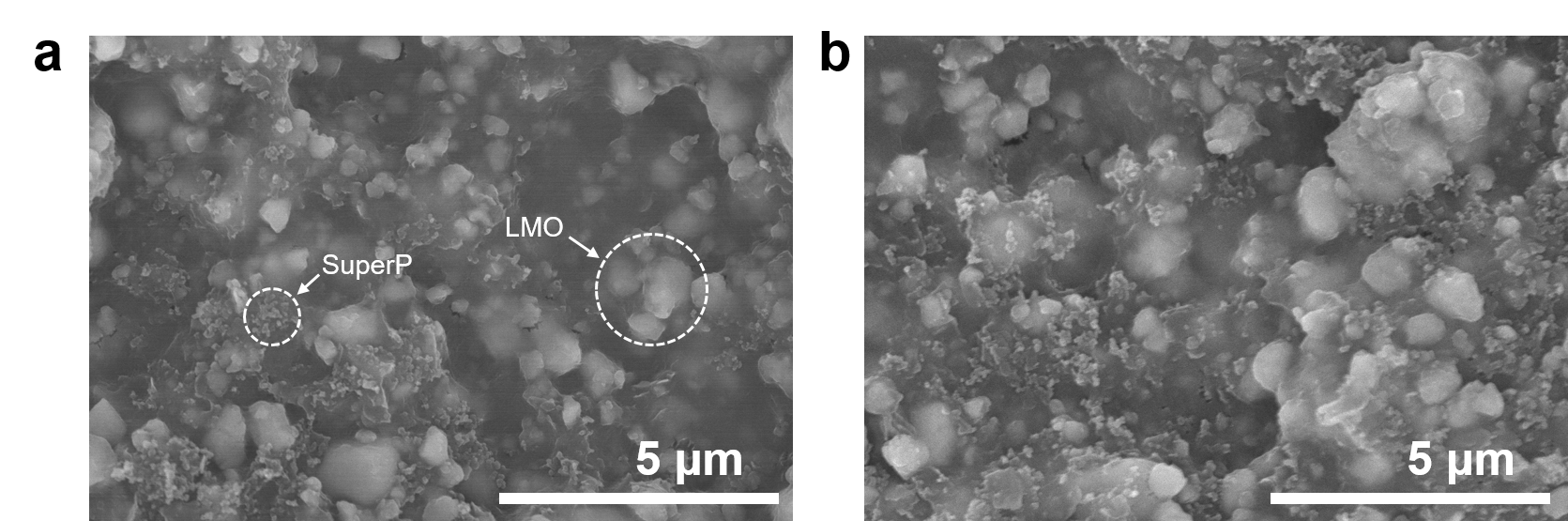


**Supplementary Figure 12.** SEM images of LMO@Gn cathodes (**a**) before and (**b**) after 200 cycles.


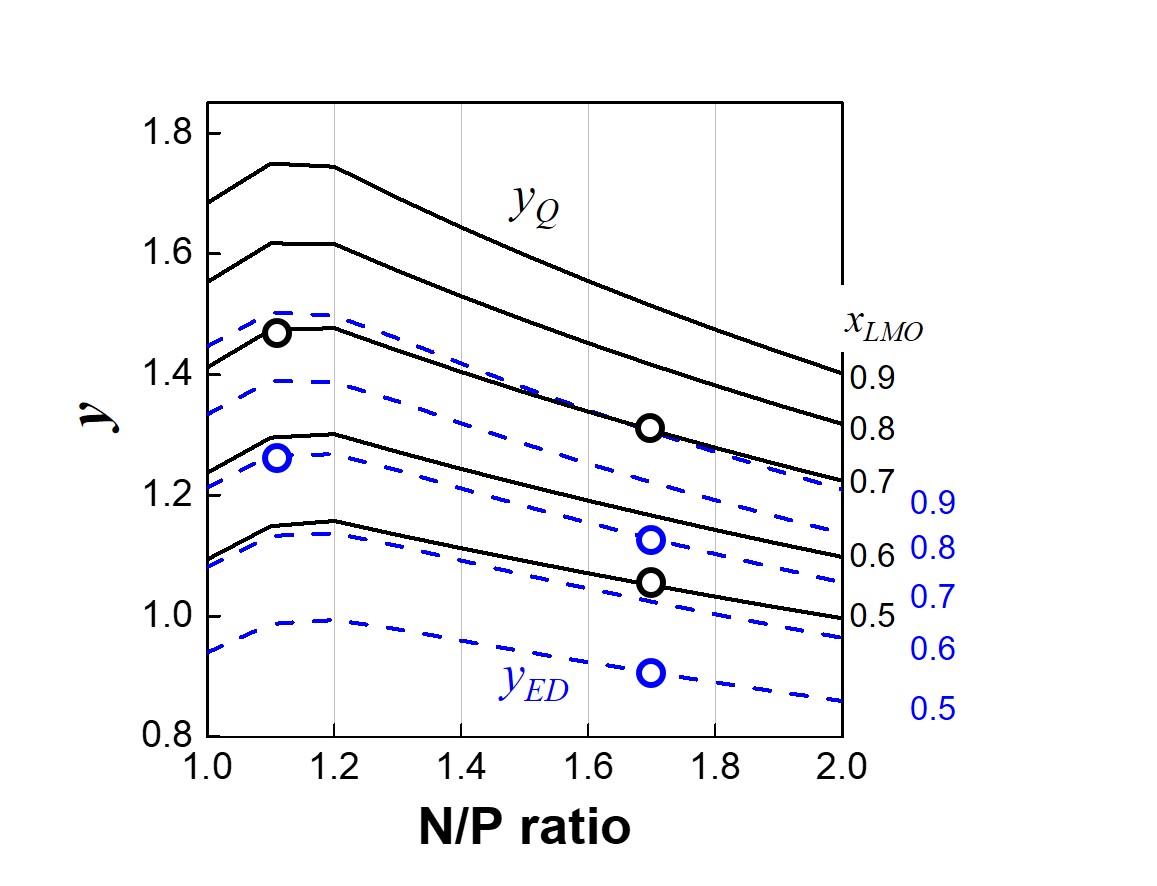


**Supplementary Figure 13.** Energy density improvement factor (y_ED_) and capacity improvement factor (y_Q_) as a function of the N/P ratio of LMO@Gn||Li*_n_*C_6_ (N/P) and the fractional content of LMO in cathodes (*x*_LMO_). LMO@Gn||Li*_n_*C_6_ at various N/P and *x*_LMO_ was compared with LMO||C_6_ at a practical value of N/P and *x*_LMO_ (N/P = 1.1 and *x*_LMO_ = 0.9). The values of N/P and *x*_LMO_ used in this work were indicated by open circles.


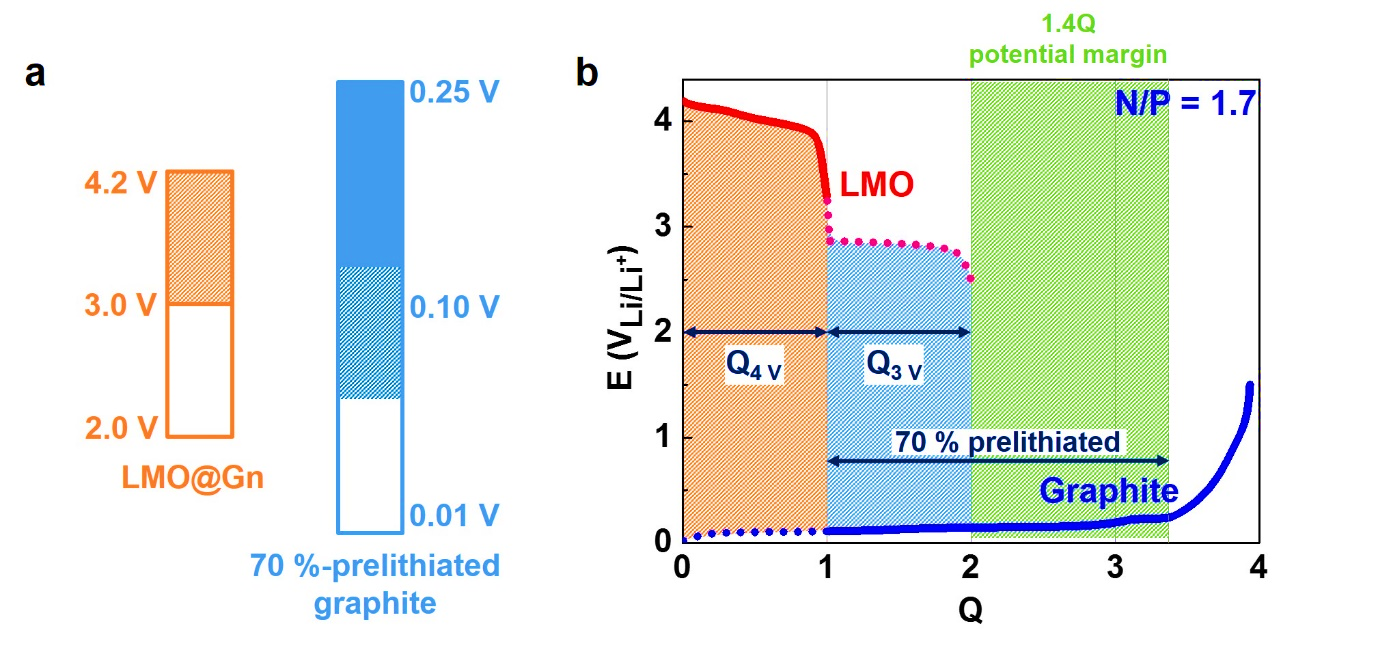


**Supplementary Figure 14. Optimized LMO@Gn||Li_n_C_6_ cell design.** (**a**) Lithium storage scheme of both cathode and anode. (**b**) Potential (*E*) profile of LMO@Gn and prelithiated graphite. The N/P ratio = 1.7.


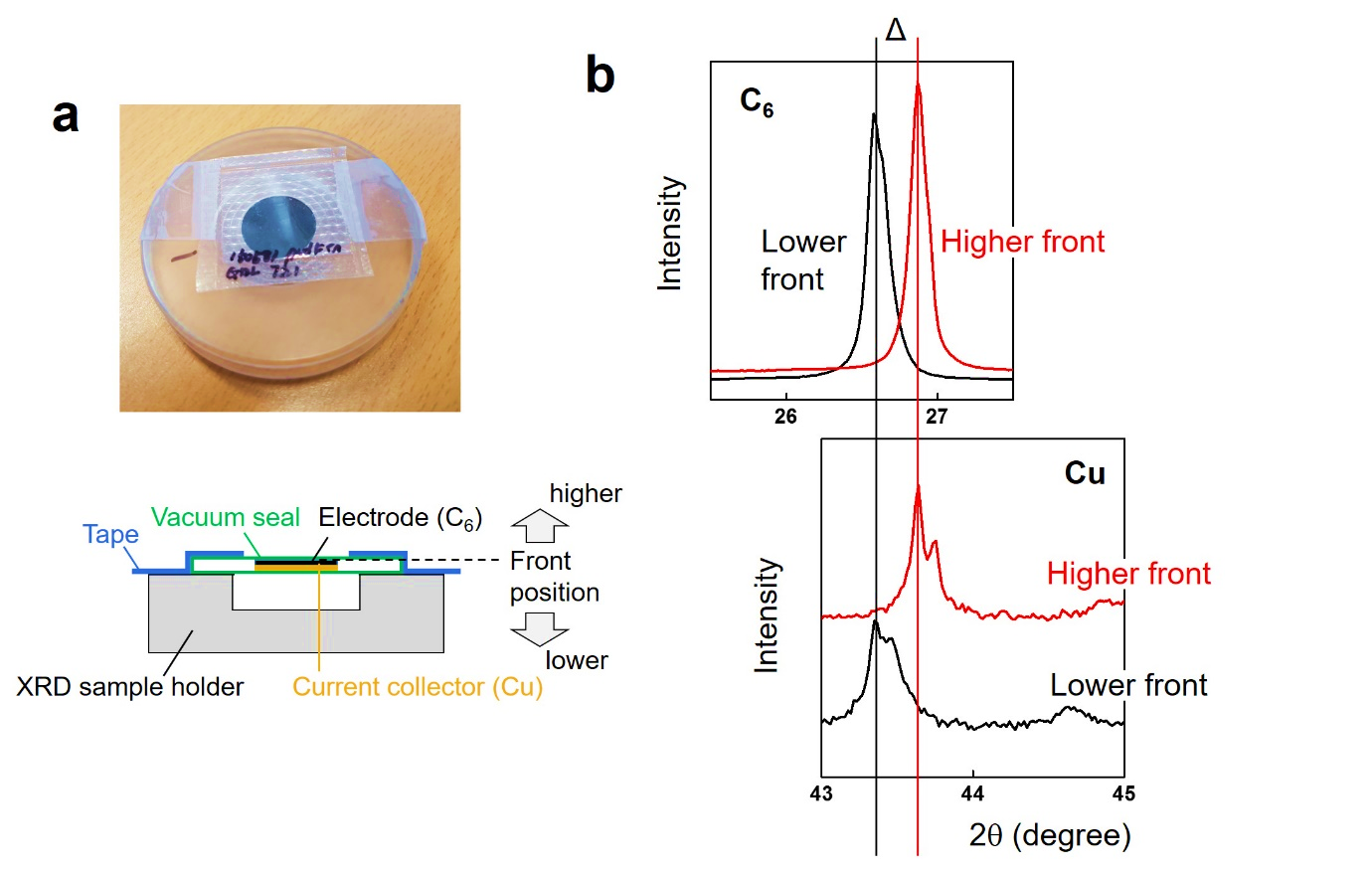


**Supplementary Figure 15.** (**a**) A hermetically sealed electrode on a XRD sample holder. Front position was indicated in the schematic. (**b**) X-ray diffraction patterns of an identical graphite sample loaded in different front positions in a sample holder. Peaks characteristic of graphite (C_6_) and Cu were positively shifted by an identical value at 0.29 degree when the sample was placed at higher position in the same sample holder.
